# Cancer and diabetes co-occurrence: A national study with 44 million person-years of follow-up

**DOI:** 10.1371/journal.pone.0276913

**Published:** 2022-11-28

**Authors:** Jason Gurney, James Stanley, Andrea Teng, Jeremy Krebs, Jonathan Koea, Chunhuan Lao, Ross Lawrenson, Ineke Meredith, Dianne Sika-Paotonu, Diana Sarfati

**Affiliations:** 1 Cancer and Chronic Conditions (C3) Research Group, Department of Public Health, University of Otago, Wellington, New Zealand; 2 Department of Medicine, University of Otago, Wellington, New Zealand; 3 Department of General Surgery, Waitemata District Health Board, Auckland, New Zealand; 4 Medical Research Centre, University of Waikato, Hamilton, New Zealand; 5 Department of Surgery, Capital and Coast District Health Board, Wellington, New Zealand; 6 Department of Pathology & Molecular Medicine, University of Otago, Wellington, New Zealand; 7 Te Aho o Te Kahu–Cancer Control Agency, Wellington, New Zealand; Flinders University, AUSTRALIA

## Abstract

The number of new cases of cancer is increasing each year, and rates of diabetes mellitus are also increasing dramatically over time. It is not an unusual occurrence for an individual to have both cancer and diabetes at the same time, given they are both individually common, and that one condition can increase the risk of the other. In this manuscript, we use national-level diabetes (Virtual Diabetes Register) and cancer (New Zealand Cancer Registry) data on nearly five million individuals over 44 million person-years of follow-up to examine the occurrence of cancer amongst a national prevalent cohort of patients with diabetes. We completed this analysis separately by cancer for the 24 most commonly diagnosed cancers in Aotearoa New Zealand, and then compared the occurrence of cancer among those with diabetes to those without diabetes. We found that the rate of cancer was highest amongst those with diabetes for 21 of the 24 most common cancers diagnosed over our study period, with excess risk among those with diabetes ranging between 11% (non-Hodgkin’s lymphoma) and 236% (liver cancer). The cancers with the greatest difference in incidence between those with diabetes and those without diabetes tended to be within the endocrine or gastrointestinal system, and/or had a strong relationship with obesity. However, in an absolute sense, due to the volume of breast, colorectal and lung cancers, prevention of the more modest excess cancer risk among those with diabetes (16%, 22% and 48%, respectively) would lead to a substantial overall reduction in the total burden of cancer in the population. Our findings reinforce the fact that diabetes prevention activities are also cancer prevention activities, and must therefore be prioritised and resourced in tandem.

## Introduction

Cancer is a leading cause of death globally and in Aotearoa New Zealand (NZ), and its prevalence will rise as our population ages [1]. There are approximately 19 million new cancer cases each year, and almost 10 million cancer deaths [2]. Similar to other regions, recent projections indicate that there will be an approximate doubling of cancer patients aged 65 years and over, in the Oceania region by 2035 [3].

Rates of diabetes mellitus (hereafter *diabetes*) are also increasing dramatically over time, and are poised to increase by approximately 100 million cases over the next ten years [4]. Rates of diabetes are increasing in Aotearoa New Zealand by a staggering 7% per year [5]. In 2018, around 253,000 New Zealanders had some form of diabetes [6], with 90–95% of these likely to be Type 2 [7].

It is not an unusual occurrence for an individual to have both cancer and diabetes at the same time, given they are both individually common, and that one condition can increase the risk of the other [8–11]. Based on international evidence, we might expect approximately 35% of the population to be diagnosed with diabetes and 44% with cancer within their lifetime; with around 15% diagnosed with both [12].

The number of people diagnosed with cancer is projected to increase substantially over the coming decades–while simultaneously, the number of people living with diabetes will increase dramatically over the same period. Diabetes and cancer co-occurrence is likely to increase the complexity of the care of both conditions–in the context of cancer, the timing of diagnosis, access to treatment, and ultimately affect survival outcomes are impacted. Patients with diabetes are more likely to have cancer that is further advanced at diagnosis, compared to those without diabetes [13–15], while those patients with diabetes and localised cancer at diagnosis are more likely to develop metastases [16]. There is also evidence indicating that patients with diabetes are less likely to receive aggressive curative treatment for their cancer [17, 18]. People with diabetes have poorer survival from cancer [19, 20] (including women with breast cancer [15, 21]), with a large meta-analysis finding that cancer patients with diabetes were 41% more likely to die than cancer patients without diabetes [22].

Given the growing issue of diabetes and cancer co-occurrence, there is a need to better understand the burden of this co-occurrence at a population level and across the spectrum of cancer types. In Aotearoa New Zealand, we are well placed to provide robust evidence on the prevalence of cancer and diabetes co-occurrence at present, and also describe how this is patterned by cancer type, due to advantages in health care funding and the collection of health data, and its centralisation. In addition to the existence of a high-quality and mandated national Cancer Registry [23], Aotearoa New Zealand has a Virtual Diabetes Register, which uses national health data to ascribe diabetes status to every individual living in Aotearoa New Zealand [24]. This data capability is relatively unique in the international setting, and in the context of this study, allows us to measure the extent to which our population experiences diabetes and cancer co-occurrence.

Therefore, in this manuscript we use national-level data on nearly five million individuals over 44 million person-years of follow-up and describe the burden of cancer and diabetes co-occurrence. We examine the occurrence of cancer amongst a national prevalent cohort of patients with diabetes, separately by cancer for the 24 most commonly diagnosed cancers in Aotearoa New Zealand, and then compare this occurrence to those without diabetes.

## Materials and methods

### Participants and data sources

We used Statistics New Zealand’s Integrated Data Infrastructure (IDI) estimated resident population (ERP) for each year between 2008–2017 as our baseline population. We extracted annual national cohorts (or ‘annual cohorts’ / ‘risk sets’), beginning in the middle of 2008 through to the middle of 2018 (i.e. 10 individual annual cohorts, from 1st July through to 30th June of the next year). Diabetes status for each annual risk set was defined at the start of each annual period using data from the Virtual Diabetes Register (VDR, see below). Cancer incidence was also determined for each annual risk set to allow calculation of incidence of cancer among those with and without diabetes (total follow-up time: 43,967,454 person-years).

The methods underlying the VDR are detailed elsewhere [25]; but in brief, an algorithm is utilised to define diabetes prevalence within the population on the basis of national level data on inpatient hospitalisation ICD diagnosis codes (using the National Minimum Dataset, or NMDS), relevant outpatient events (National Non-Admitted Patient Collection, NNPAC), retinal screening (using regional retinal screening programme datasets), pharmaceutical dispensing (Pharmaceutical Claims dataset) and pathology test claims (Laboratory Claims dataset). The algorithm has been iteratively modified over time to improve sensitivity (around 90%) and specificity (around 96%), and has been validated against primary care registers [25, 26]. The VDR is used to determine official diagnosed diabetes prevalence in Aotearoa New Zealand, but does not differentiate between diabetes type [24].

Once we had set our annual cohorts and defined diabetes status, we utilised the IDI to link the cohorts to the New Zealand Cancer Registry (NZCR; follow-up period 2008–2018). The NZCR is a nationally-mandated population-based registry of all malignancies diagnosed in Aotearoa New Zealand, with the exception of basal and squamous cell skin cancers [23]. We initially searched for all malignancies (i.e. all ‘C’ codes within the International Classification of Diseases [ICD]-10-AM coding definitions [27]), and identified the 24 most commonly diagnosed cancers amongst the total cohort over the study period.

### Variables

As noted above, **diabetes status** (with diabetes/without diabetes) was derived from the VDR, while **cancer status** was derived from the NZCR. **Cancer type** (e.g. lung cancer) was determined using site codes on the NZCR. **Date of cancer diagnosis** was derived from the NZCR. **Age** was determined as age at the start of the individual annual cohort, and derived from the IDI personal details table. Age was divided into seven categories (<20, 20–29, 30–39, 40–49, 50–59, 60–69, 70+ years) for the purposes of age-standardisation. **Sex** (male/female) was also defined using the IDI personal details table.

### Statistical analysis

The statistical analysis proceeded by constructing annual cohorts (defined above) which were then summed to provide person-time at risk across the entire study period. This approach was chosen to allow for handling of (a) censoring of follow-up time and (b) changes in diabetes status across the study period (time-varying covariate: diabetes status defined at the start of each annual cohort). These choices also reflected restrictions in the data available for analysis (see *Discussion*).

In terms of censoring, we utilised the estimated residential population to determine who is at risk using a constrained follow-up period. At the beginning of each mid-year cycle, the ERP removes those who died or emigrated during the previous 12 months, leaving just the numbers of those at risk in the subsequent period for our annual cohorts.

We calculated cancer incidence rates for both diabetes groups (with diabetes/without diabetes). The numerator for rate calculation was the number of cancers (in total, and by cancer type) occurring among patients with and without diabetes, both in total and stratified by sex. Because we wanted to calculate total cancer incidence, it was possible for an individual to have a number of different cancer types within each year, and the same individual could also end up with a second primary cancer of the same type in a subsequent year. The denominator was the number of person-years of follow-up time among patients with and without diabetes, in total and stratified by sex. Person-time of follow-up was calculated by summing the number of years of follow-up within each diabetes group (with/without diabetes) for each annual cohort, and then summing that time across all years of follow-up.

Rates of cancer incidence were age standardised using direct standardisation methods. Because rates of diabetes are disproportionately high amongst Māori (and Pacific) peoples in Aotearoa New Zealand [5], and because these populations have a substantially younger age structure than the non-Māori/non-Pacific population [28], we utilised the total Māori diabetes population over the study period as the standard. Finally, crude and age-standardised rate ratios (RRs) comparing the rate of cancer between those with and without diabetes were calculated using the age-standardised rates to be consistent in reporting ratios that were directly derived from the reported age-standardised rates (rather than age-adjusted RRs from regression modelling). RRs are presented with their 95% confidence intervals (CIs).

Data analysis was completed in SAS Enterprise Guide v7.1 and SAS v9.4 (SAS Institute Inc., USA), and Microsoft Excel (Microsoft Corporation, USA). Ethical approval for this study was sought and received from the University of Otago Human Ethics Committee (reference # HD21/073). All data were de-identified prior to being made available by the Ministry of Health, and our ethical approval did not require informed consent for this retrospective analysis of national-level data.

## Results

### Overall trends

**Fig 1:**
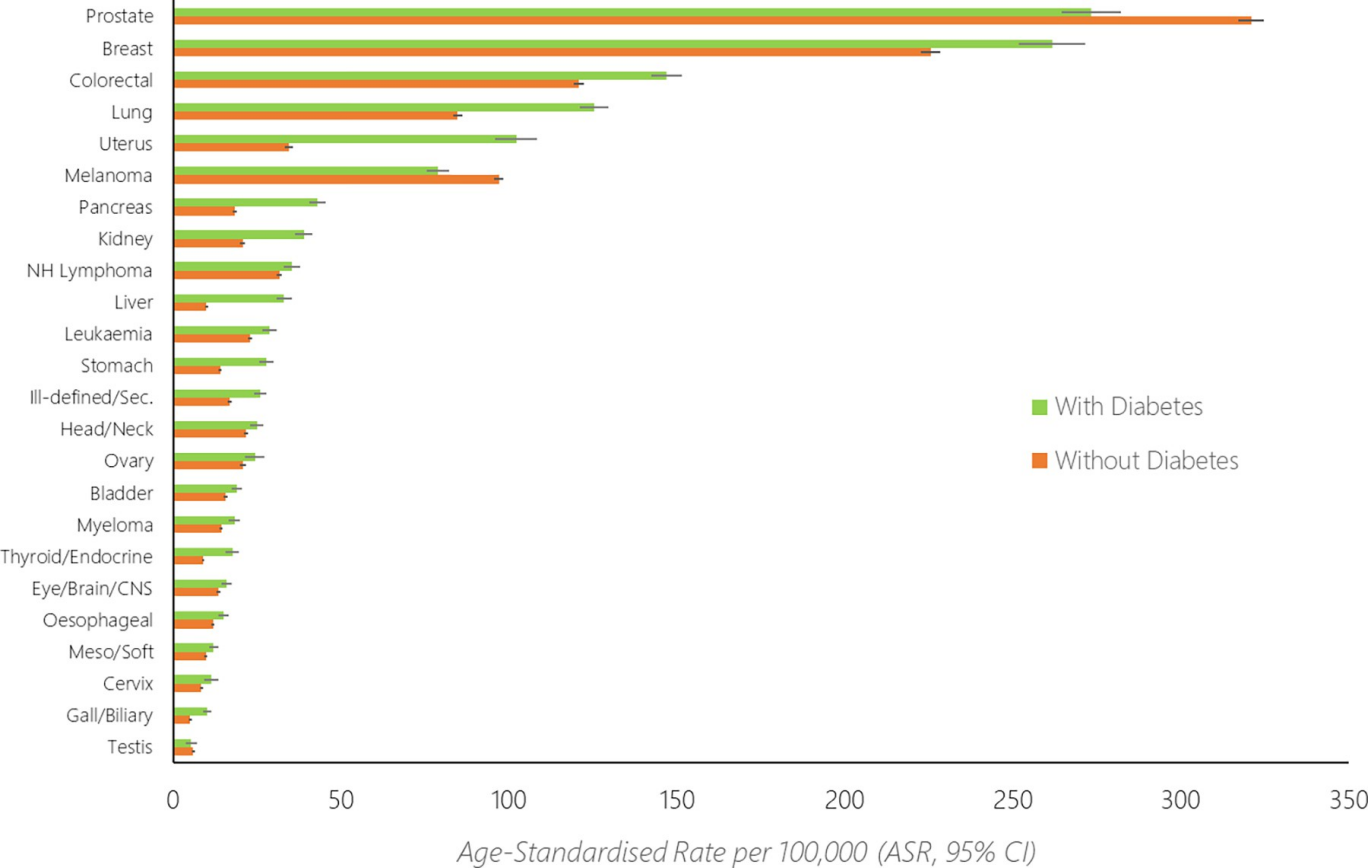
Age–standardised rates (ASR, 95% confidence intervals) of cancer amongst those with and without diabetes, for the 24 most commonly diagnosed cancers in Aotearoa New Zealand.

**Fig 2:**
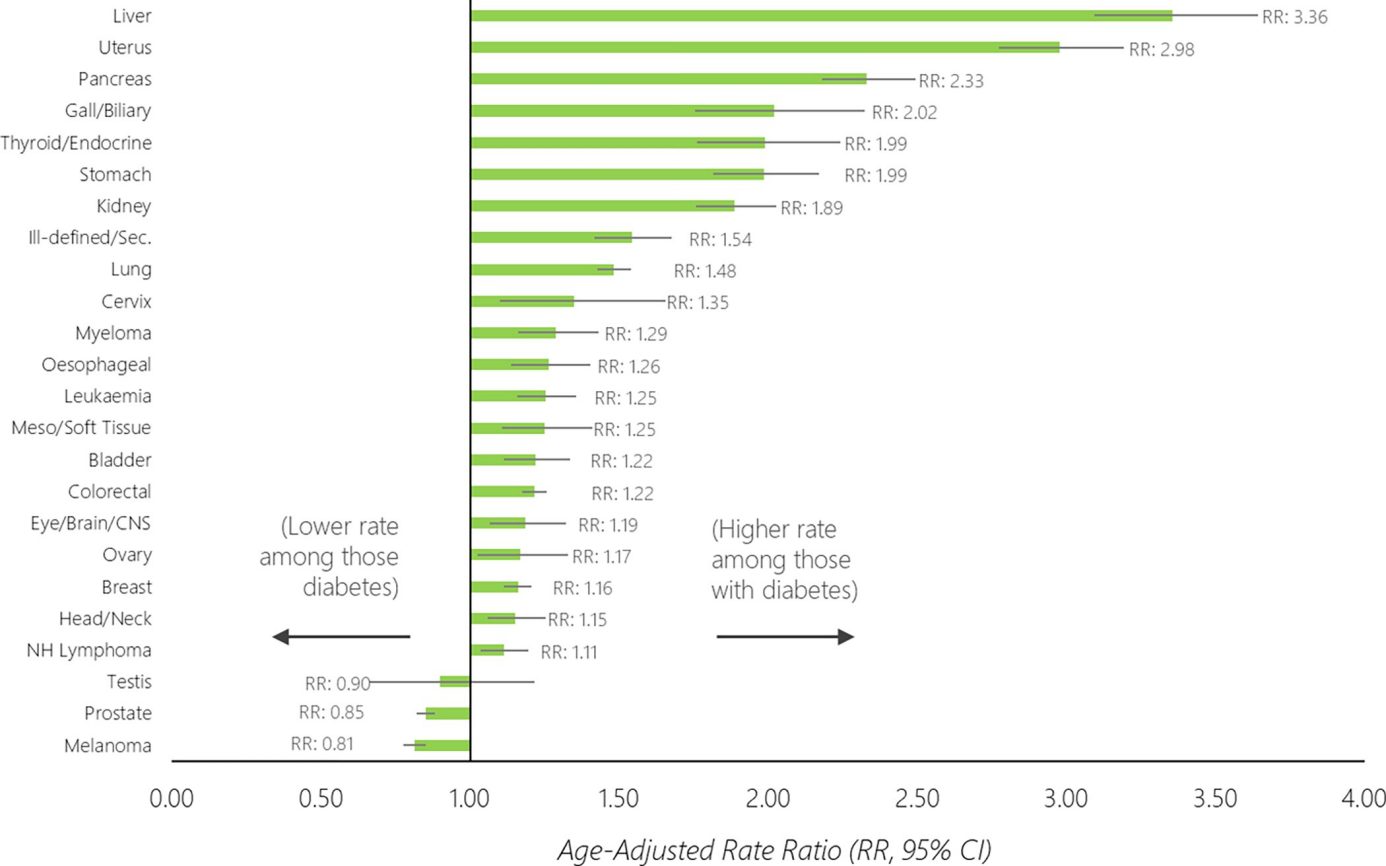
Age–standardised rate ratios (RR) of cancer between those with diabetes compared to those without diabetes (reference group), for the 24 most commonly diagnosed cancers in Aotearoa New Zealand.

Age-standardised rates of cancer incidence among those with and without diabetes are shown in **Fig 1**, with the base data for the figure shown in S1 Table. Rate ratios comparing the rate of cancer incidence between the two groups are shown in **Fig 2**, with the base data for the figure shown in S2 Table. Overall, 176,055 individuals without diabetes developed cancer over the study period (age-standardised rate [ASR]: 875/100,000, 95% CI 870–879), along with 31,155 individuals with diabetes (ASR: 1,097/100,000, 1,084–1,111). When standardised for age, the rate of cancer was 25% higher among those with diabetes when compared to those without (age-standardised rate ratio [RR]: 1.25, 95% CI 1.24–1.27).

The age-standardised rate (**Figs 1** and **2**) was highest for prostate (ASR: without diabetes, 321/100,000; with diabetes, 273/100,000), breast (ASR: without diabetes: 226/100,000, with diabetes 262/100,000), colorectal (ASR: without diabetes, 121/100,000; with diabetes 147/100,000), lung (ASR: without diabetes, 85/100,000; with diabetes, 125/100,000) and uterine cancers (ASR: without diabetes, 34/100,000; with diabetes 102/100,000). The relative difference in rates between those with diabetes compared to without diabetes was highest for liver (RR: 3.36, 95% CI 3.09–3.64), uterine (RR: 2.98, 95% CI 2.77–3.19), pancreatic (RR: 2.33, 95% CI 2.18–2.49), gallbladder/biliary (RR: 2.02, 95% CI 1.76–2.32) and thyroid/endocrine cancers (RR: 1.99, 95% CI 1.79–2.24). The cancer incidence rate was higher amongst those with diabetes compared to those without diabetes for 21 of the 24 investigated cancers (**Figs 1 and 2**).

### Trends by sex

**Fig 3:**
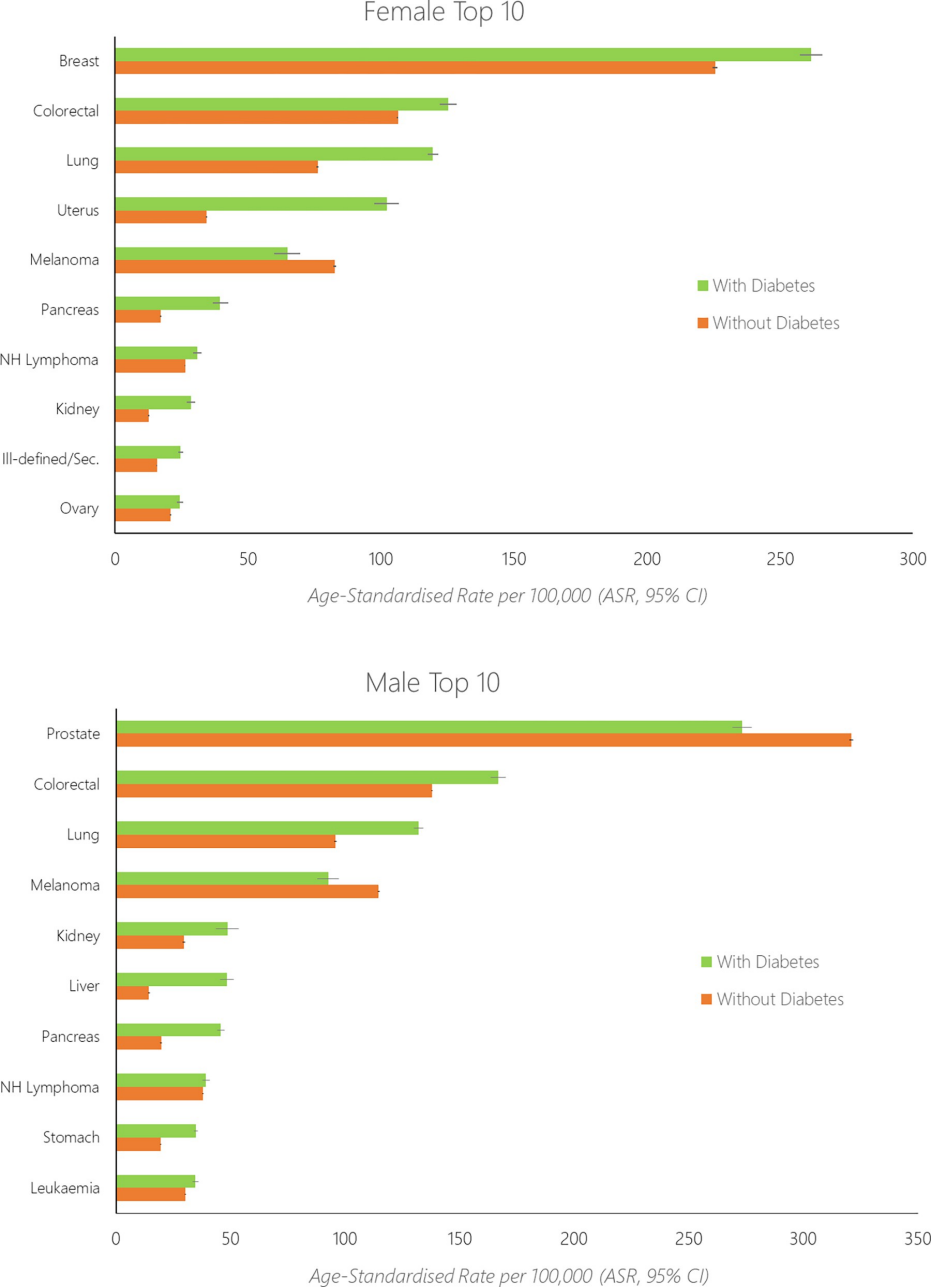
Age–standardised rates (ASR) of the top–10 cancers diagnosed among females (top) with diabetes and males (bottom) with diabetes, alongside data for those without diabetes.

**Fig 4:**
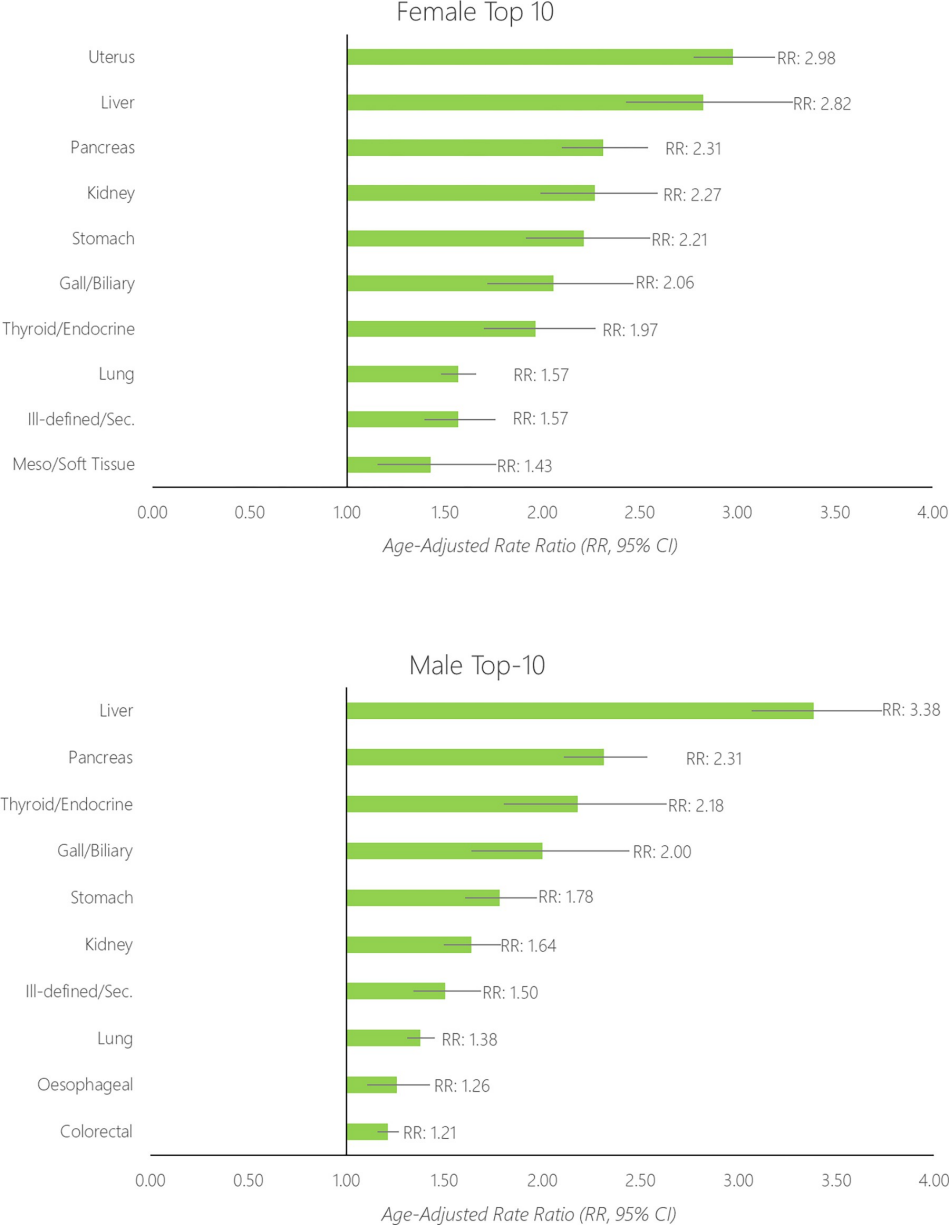
Age–standardised rate ratio (RR) of cancer between those with diabetes compared to those without diabetes (reference group), separately for the top–10 largest disparities for females (top) and males (bottom).

Age-standardised rates for the top-10 most common cancers among females and males with diabetes are shown in **Fig 3** alongside data for those without diabetes, while rates for the full 24 cancers are shown in S3 Table. Sex-stratified rate ratios comparing the rate of cancer incidence between the two diabetes groups are shown in **Fig 4**.

For females, overall the age-standardised rate of cancer was 761/100,000 individuals without diabetes, and 1,037/100,000 individuals with diabetes. The rate of cancer was 36% higher among females with diabetes compared to those without (age-standardised rate ratio [RR]: 1.36, 95% CI 1.34–1.39). The age-standardised rate was highest for breast (ASR: without diabetes, 226/100,000; with diabetes, 262/100,000), colorectal (ASR: without diabetes: 106/100,000, with diabetes 125/100,000), lung (ASR: without diabetes, 76/100,000; with diabetes 119/100,000), uterine (ASR: without diabetes, 34/100,000; with diabetes, 102/100,000) and melanoma cancers (ASR: without diabetes, 83/100,000; with diabetes 65/100,000). For females the relative difference in rate between those with diabetes compared to without diabetes was highest for uterine (RR: 2.98, 95% CI 2.77–3.19), liver (RR: 2.82, 95% CI 2.43–3.28), pancreatic (RR: 2.31, 95% CI 2.10–2.54), kidney (RR: 2.27, 95% CI 1.99–2.59) and stomach cancers (RR: 2.21, 95% CI 1.92–2.55).

For males, overall the age-standardised rate of cancer was 1,015/100,000 individuals without diabetes, and 1,156/100,000 individuals with diabetes. The rate of cancer was 14% higher among males with diabetes compared to those without (age-standardised rate ratio [RR]: 1.14, 95% CI 1.12–1.16). The age-standardised rate was highest for prostate (ASR: without diabetes, 321/100,000; with diabetes, 273/100,000), colorectal (ASR: without diabetes: 138/100,000, with diabetes 167/100,000), lung (ASR: without diabetes, 96/100,000; with diabetes 132/100,000), melanoma (ASR: without diabetes, 115/100,000; with diabetes, 93/100,000) and kidney cancers (ASR: without diabetes, 30/100,000; with diabetes 49/100,000). For males the relative difference in rate between those with diabetes compared to without diabetes was highest for liver (RR: 3.38, 95% CI 3.07–3.73), pancreatic (RR: 2.31, 95% CI 2.11–2.53), thyroid/endocrine (RR: 2.18, 95% CI 1.80–2.63), gallbladder/biliary tract (RR: 2.00, 95% CI 1.64–2.45) and stomach cancers (RR: 1.78, 95% CI 1.61–1.97).

Of the female-specific cancers, the strongest relative difference in cancer incidence between those with diabetes compared to those without diabetes, was found for uterine cancers (RR ~3). The rate of cervical cancers were approximately 35% higher among those with diabetes, and around 15% higher for both ovarian and breast cancers. Of the male-specific cancers, there was no difference in the rate of testicular cancer between those with diabetes and those without diabetes (RR: 0.90, 95% CI 0.66–1.22). The rate of prostate cancer was 15% lower among those with diabetes compared to those without (RR: 0.85, 95% CI 0.82–0.88).

## Discussion

In this study, we utilised national prevalence data on diabetes alongside national incidence data on cancer to describe the co-occurrence of these conditions, and report the extent to which some cancers occur more commonly amongst those with diabetes, when compared to those without. We found that the cancers with the highest incidence rate among those with diabetes largely reflected the most commonly diagnosed cancers in the general population. For women, the three most commonly diagnosed cancers amongst those with diabetes were breast, colorectal and lung cancers, while for men it was prostate, colorectal and lung cancers.

We found that the rate of cancer is highest amongst those with diabetes for nearly all of the 24 most common cancers diagnosed over our study period. Only melanoma, prostate and testis cancers were more common amongst those without diabetes when compared to those with diabetes. The cancers with the greatest relative difference in incidence by diabetes status tended to be within the endocrine or gastrointestinal system (liver, pancreas, gallbladder, thyroid/endocrine, stomach), and/or had a strong relationship with obesity (uterus, kidney). This trend was observable for both males and females. These findings largely echo those of previous studies, which have found similarly elevated risk of cancer among those with diabetes as we have identified here (e.g. [29–34]).

These differences in cancer burden by diabetes status have multifactorial causes. Diabetes and cancer co-occur either because a) of shared risk factors, such as socioeconomic deprivation, obesity, and physical inactivity [35–38]; b) because of direct causation between diabetes and a given cancer [8–10, 39], or vice-versa [11]; and c) because both conditions occur relatively commonly in the population. For example, the patterning of elevated risk of gastrointestinal and endocrine tumours amongst those with diabetes supports existing evidence of a strong relationship between diabetes and these tumours, likely through shared risk factors but also (particularly in the case of pancreatic cancer) a direct causative relationship [32, 40–42]. The substantially higher rate of uterine cancer amongst those with diabetes is a reminder of the importance of addressing obesity (i.e. a shared risk factor) in reducing the occurrence of both Type-2 diabetes and obesity-related cancer, including uterine, breast and steatohepatitic liver cancer [43, 44]. It must be noted that the current study is not able to disentangle whether the increased risk of some cancers among those with diabetes is a direct consequence of their diabetes, or their diabetes is a direct consequence of their cancer, or due to some other reason. In this Discussion section we focus primarily on the ramifications of this increased risk. Because the vast majority of Type-2 diabetes cases are preventable [45], we might view the excess risk of most cancers experienced by these people as also being potentially preventable. In other words, for those cancers where diabetes is linked to tumour development either directly (as a risk factor itself) or indirectly (through shared risk factors, such as obesity), the prevention of Type-2 diabetes can be viewed as also preventative against the excess cases of cancer observed among those with diabetes in our study. Prevention of these cancers would perhaps have the most dramatic impact on those cancers with the starkest relative difference between those with and without diabetes–such as liver cancer, where incidence was 236% higher amongst those with diabetes compared to those without. However, in an absolute sense, due to the volume of breast, colorectal and lung cancers, prevention of the more modest excess cancer risk amongst those with diabetes (16%, 22% and 48%, respectively), would lead to a substantial overall reduction in the total burden of cancer in the population. This observation reinforces the fact that diabetes and obesity prevention activities are also cancer prevention activities, and must therefore be prioritised and resourced in tandem.

Further, the heightened risk of cancer amongst those with diabetes provides reasoning for this group to be considered prime candidates for cancer surveillance [30]. The increased contact with health care services for diabetes management presents a prime opportunity for these services to engage in evidence-based early cancer detection activities, including the promotion of national cancer screening programmes and a low threshold for primary care referral to secondary diagnostics when a patient presents with symptoms that may be either diabetes-derived or signs of cancer. The importance of this opportunity is underpinned by evidence that cancer patients with diabetes have poorer cancer-specific survival than cancer patients without diabetes [46–48]. Unfortunately, international evidence suggests that access to cancer screening and other surveillance amongst those with diabetes is either the same or poorer than those without diabetes [49, 50], suggesting we are missing valuable opportunities for early detection of cancers amongst patients with diabetes.

Our observation of substantially lower rates of prostate cancer amongst men with diabetes compared to men without diabetes, echoes similar findings from other studies [51–54]. It is thought that detection bias may be an important driver of this observation [51], in which increased access to prostate specific antigen (PSA) screening amongst those without diabetes may increase the number of prostate cancers diagnosed among this group.

Our observation of substantially lower rates of melanoma amongst those with diabetes, is somewhat contrary to other studies which have shown either heightened risk or no additional risk relative to those without diabetes [55, 56]. Our contrary observation may be related to the profile of patients diagnosed with diabetes in Aotearoa New Zealand, which is quite different to the profile of those diagnosed with melanoma skin cancer–wherein diabetes is much more common among Māori, Pacific and South Asian peoples than European peoples [6], and higher amongst those living in socioeconomic deprivation than for those not living in deprivation [57], whereas the opposite is true for the incidence of melanoma [58, 59]. The rarity of melanoma amongst these ethnic groups and/or those living in deprivation is likely to reduce the overall observed burden of melanoma among those with diabetes when data for all ethnic groups are pooled together. Ethnic disparities in diabetes and cancer co-occurrence are the focus of a separate study that we are currently undertaking with the data sources utilised for the current study, and we will aim to explore the relationship between diabetes and melanoma there.

### Strengths and limitations

This national study utilised the total Aotearoa New Zealand population as the base, a relatively unique national dataset to determine diabetes status for all Aotearoa New Zealanders, and a national cancer register to determine cancer incidence. As such, our findings are generalizable to the total population of Aotearoa New Zealand and similar countries worldwide. Rather than studying new exposure to diabetes, diabetes status was defined based on a prevalent basis for each annual cohort, which may result in a bias in situations where the risk of an outcome is somewhat dependent on the duration of the exposure [60, 61]. Our annualised cohort approach, where diabetes exposure was reset each year, may have reduced the likelihood of this bias from occurring. Relatedly, the analysis approach of using annualised cohorts to analyse rates and rate ratios was partly dictated by these and other limitations of the available data (e.g. population data only updated on an annual basis), but closely aligns with a population-level analysis of cancer outcomes amongst those with diabetes, rather than an individual-level analysis of cancer outcomes (and their timings relative to diabetes onset) that might have been feasible if accurate diabetes onset/diagnosis information had been available.

We note that our study describes the co-occurrence of diabetes and cancer in purely descriptive terms, and does not take into account the mediating and/or confounding impact of other chronic conditions (including obesity) on cancer risk, nor the role of social determinants of health such as socioeconomic status, which limits our ability to make causal inferences regarding the relationship between the two conditions. Relatedly, while it is thought that drugs used to treat cancer may either cause diabetes or worsen pre-existing diabetes [62], the current study was designed to detect new cases of cancer amongst those already with diabetes, meaning that we were unable to examine reverse causation (i.e. the development of diabetes following a diagnosis of cancer). Finally, we could not differentiate between Type 1 and Type 2 diabetes using the available data; and the available data only recorded sex as male or female.

## Conclusions

In this national study of nearly five million individuals and 44 million person-years of follow-up, we found that the cancers with the highest incidence rate amongst those with diabetes largely reflected the cancers most commonly diagnosed within the general population. We found that the rate of cancer was highest amongst those with diabetes for 21 of the 24 most common cancers diagnosed over our study period, with excess risk among those with diabetes ranging between 11% (non-Hodgkin’s lymphoma) and 236% (liver cancer). The cancers with the greatest difference in incidence between those with diabetes and those without, tended to be within the endocrine or gastrointestinal system, and/or had a strong relationship with obesity. However, in an absolute sense, due to the volume of breast, colorectal and lung cancers, prevention of the more modest excess cancer risk among those with diabetes (16%, 22% and 48%, respectively) would lead to a substantial overall reduction in the total burden of cancer in the population. Our findings reinforce the fact that diabetes prevention activities are also cancer prevention activities, and must therefore be prioritised and resourced in tandem.

## Supporting information

S1 Table Table of age-standardised rates (ASR) of cancer among those with and without diabetes, for the 24 most commonly diagnosed cancers in Aotearoa New Zealand. (DOCX)

S2 Table Table of age-standardised rate ratios (RR) of the rate of cancer between those with diabetes compared to those without diabetes (reference group), for the 24 most commonly diagnosed cancers in Aotearoa New Zealand. (DOCX)

S3 Table Table of age-standardised rates (ASR) of cancer among those with and without diabetes, for the most commonly diagnosed cancers in Aotearoa New Zealand, for a) females and b) males. PY = person-years.
(DOCX)

S4 Table Table of age-standardised rate ratios (RR) of the rate of cancer between those with diabetes compared to those without diabetes (reference group), for the most commonly diagnosed cancers in Aotearoa New Zealand, for a) females and b) males. (DOCX)
