# Supplementary material for: Cancer and diabetes co-occurrence: A national study with 44 million person-years of follow-up
Source: PLoS One. 2022 Nov 28;17(11):e0276913. doi: 10.1371/journal.pone.0276913 (PMC9704677; doi:10.1371/journal.pone.0276913)
Supplement: S1 Table — (DOCX) [file pone.0276913.s001.docx]

**S1 Table:** Table of age-standardised rates (ASR) of cancer among those with and without diabetes, for the 24 most commonly diagnosed cancers in Aotearoa New Zealand.

|  | **Without Diabetes** | | | **With Diabetes** | | |
| --- | --- | --- | --- | --- | --- | --- |
|  |  | Cancer Rate (n/100,000 PY) | |  | Cancer Rate (n/100,000 PY) | |
|  | *n* | *Crude* | *Age Std.* | *n* | *Crude* | *Age Std.* |
| **Total** | 176,055 | 421.6 | 874.5 (870.3-878.7) | 31,155 | 1411.9 | 1097.4 (1084.3-1110.5) |
|  |  |  |  |  |  |  |
| **By Cancer** |  |  |  |  |  |  |
| *Prostate* | 26,694 | 63.9 | 321 (317.2-324.9) | 4,068 | 184.4 | 273.3 (264.5-282.1) |
| *Myeloma* | 2,724 | 6.5 | 14.2 (13.6-14.7) | 531 | 24.1 | 18.3 (16.6-19.9) |
| *Breast* | 25,068 | 60.0 | 225.6 (222.6-228.5) | 3,105 | 140.7 | 261.7 (251.8-271.6) |
| *Colorectal* | 23,664 | 56.7 | 120.8 (119.3-122.4) | 4,563 | 206.8 | 147 (142.4-151.6) |
| *Lung* | 15,978 | 38.3 | 84.6 (83.3-86) | 3,762 | 170.5 | 125.4 (121.2-129.7) |
| *Uterus* | 3,567 | 8.5 | 34.3 (33.2-35.5) | 1,116 | 50.6 | 102.1 (95.8-108.4) |
| *Melanoma* | 20,178 | 48.3 | 97 (95.6-98.4) | 2,307 | 104.5 | 78.8 (75.3-82.2) |
| *Pancreas* | 3,564 | 8.5 | 18.4 (17.8-19) | 1,311 | 59.4 | 42.9 (40.4-45.4) |
| *Kidney* | 4,107 | 9.8 | 20.6 (19.9-21.2) | 1,011 | 45.8 | 38.9 (36.3-41.5) |
| *NH Lymphoma* | 6,465 | 15.5 | 31.6 (30.8-32.4) | 1,002 | 45.4 | 35.2 (32.9-37.6) |
| *Liver* | 1,944 | 4.7 | 9.8 (9.4-10.3) | 915 | 41.5 | 32.9 (30.6-35.2) |
| *Leukaemia* | 5,184 | 12.4 | 22.9 (22.2-23.6) | 819 | 37.1 | 28.7 (26.5-30.8) |
| *Stomach* | 2,808 | 6.7 | 13.9 (13.3-14.4) | 783 | 35.5 | 27.6 (25.5-29.7) |
| *Ill-defined/Sec.* | 3,312 | 7.9 | 16.8 (16.2-17.4) | 852 | 38.6 | 25.9 (24.1-27.8) |
| *Head/Neck* | 4,365 | 10.5 | 21.6 (20.9-22.3) | 663 | 30.0 | 24.9 (22.9-26.9) |
| *Ovary* | 2,334 | 5.6 | 20.8 (19.9-21.7) | 306 | 13.9 | 24.3 (21.3-27.2) |
| *Bladder* | 2,988 | 7.2 | 15.5 (14.9-16.1) | 645 | 29.2 | 18.9 (17.4-20.5) |
| *Thyroid/Endocrine* | 2,427 | 5.8 | 8.8 (8.4-9.2) | 366 | 16.6 | 17.5 (15.6-19.4) |
| *Eye/Brain/CNS* | 3,114 | 7.5 | 13.4 (12.8-13.9) | 405 | 18.4 | 15.9 (14.2-17.5) |
| *Oesophageal* | 2,253 | 5.4 | 11.8 (11.4-12.3) | 459 | 20.8 | 14.9 (13.4-16.4) |
| *Meso/Soft Tissue* | 2,097 | 5.0 | 9.6 (9.2-10.1) | 327 | 14.8 | 12 (10.6-13.4) |
| *Cervix* | 1,332 | 3.2 | 8.3 (7.8-8.9) | 114 | 5.2 | 11.2 (9.1-13.4) |
| *Gall/Biliary* | 966 | 2.3 | 5 (4.7-5.4) | 300 | 13.6 | 10.1 (8.9-11.3) |
| *Testis* | 1,416 | 3.4 | 5.9 (5.5-6.3) | 45 | 2.0 | 5.3 (3.6-6.9) |
