# Supplementary material for: Cancer and diabetes co-occurrence: A national study with 44 million person-years of follow-up
Source: PLoS One. 2022 Nov 28;17(11):e0276913. doi: 10.1371/journal.pone.0276913 (PMC9704677; doi:10.1371/journal.pone.0276913)
Supplement: S2 Table — (DOCX) [file pone.0276913.s002.docx]

**S2 Table:** Table of age-adjusted rate ratios (RR) of the rate of cancer between those with diabetes compared to those without diabetes (reference group), for the 24 most commonly diagnosed cancers in Aotearoa New Zealand.

|  | **Without Diabetes** | **With Diabetes** | |
| --- | --- | --- | --- |
|  |  | Cancer Rate Ratio (95% CI) | |
|  |  | *Crude* | *Adj.* |
| **Total** | Reference | 3.4 | 1.25 (1.24-1.27) |
|  |  |  |  |
| **By Cancer** |  |  |  |
| *Liver* | Reference | 8.9 | 3.36 (3.09-3.64) |
| *Uterus* | Reference | 5.9 | 2.98 (2.77-3.19) |
| *Pancreas* | Reference | 7.0 | 2.33 (2.18-2.49) |
| *Gall/Biliary* | Reference | 5.9 | 2.02 (1.76-2.32) |
| *Stomach* | Reference | 5.3 | 1.99 (1.82-2.17) |
| *Thyroid/Endocrine* | Reference | 2.9 | 1.99 (1.76-2.24) |
| *Kidney* | Reference | 4.7 | 1.89 (1.76-2.03) |
| *Ill-defined/Sec.* | Reference | 4.9 | 1.54 (1.42-1.68) |
| *Lung* | Reference | 4.5 | 1.48 (1.43-1.54) |
| *Cervix* | Reference | 1.6 | 1.35 (1.1-1.66) |
| *Myeloma* | Reference | 3.7 | 1.29 (1.16-1.43) |
| *Oesophageal* | Reference | 3.9 | 1.26 (1.14-1.4) |
| *Leukaemia* | Reference | 3.0 | 1.25 (1.16-1.36) |
| *Meso/Soft Tissue* | Reference | 3.0 | 1.25 (1.11-1.41) |
| *Bladder* | Reference | 4.1 | 1.22 (1.11-1.34) |
| *Colorectal* | Reference | 3.6 | 1.22 (1.18-1.26) |
| *Eye/Brain/CNS* | Reference | 2.5 | 1.19 (1.07-1.32) |
| *Ovary* | Reference | 2.5 | 1.17 (1.03-1.33) |
| *Breast* | Reference | 2.3 | 1.16 (1.11-1.21) |
| *Head/Neck* | Reference | 2.9 | 1.15 (1.06-1.25) |
| *NH Lymphoma* | Reference | 2.9 | 1.11 (1.04-1.2) |
| *Testis* | Reference | 0.6 | 0.9 (0.66-1.22) |
| *Prostate* | Reference | 2.9 | 0.85 (0.82-0.88) |
| *Melanoma* | Reference | 2.2 | 0.81 (0.78-0.85) |

Adj. = age-standardised rate ratio.
