# Supplementary material for: Cancer and diabetes co-occurrence: A national study with 44 million person-years of follow-up
Source: PLoS One. 2022 Nov 28;17(11):e0276913. doi: 10.1371/journal.pone.0276913 (PMC9704677; doi:10.1371/journal.pone.0276913)
Supplement: S3 Table — PY = person-years. (DOCX) [file pone.0276913.s003.docx]

**S3 Table:** Table of age-standardised rates (ASR) of cancer among those with and without diabetes, for the most commonly diagnosed cancers in Aotearoa New Zealand, for a) females and b) males. PY = person-years.

a)

|  | **Female** | | | | | |
| --- | --- | --- | --- | --- | --- | --- |
|  | **Without Diabetes** | | | **With Diabetes** | | |
|  |  | Cancer Rate (n/100,000 PY) | |  | Cancer Rate (n/100,000 PY) | |
|  | *n* | *Crude* | *Age Std.* | *n* | *Crude* | *Age Std.* |
| **Total** | 84,798 | 402 | 761.3 (756-766.7) | 13,809 | 1,264 | 1037.1 (1018.2-1055.9) |
|  |  |  |  |  |  |  |
| **By Cancer** |  |  |  |  |  |  |
| *Breast* | 25,068 | 118.8 | 225.6 (222.6-228.5) | 3,105 | 284.2 | 261.7 (251.8-271.6) |
| *Colorectal* | 11,484 | 54.4 | 106.3 (104.3-108.3) | 1,956 | 179.0 | 125.3 (119.3-131.4) |
| *Lung* | 7,749 | 36.7 | 76.1 (74.4-77.9) | 1,695 | 155.1 | 119.4 (113.2-125.5) |
| *Uterus* | 3,567 | 16.9 | 34.3 (33.2-35.5) | 1,116 | 102.2 | 102.1 (95.8-108.4) |
| *Melanoma* | 9,453 | 44.8 | 82.6 (80.8-84.3) | 882 | 80.7 | 64.7 (60-69.3) |
| *Pancreas* | 1,830 | 8.7 | 17.1 (16.3-17.9) | 621 | 56.8 | 39.5 (36.1-42.9) |
| *NH Lymphoma* | 2,853 | 13.5 | 26.3 (25.3-27.3) | 432 | 39.5 | 30.9 (27.7-34) |
| *Kidney* | 1,386 | 6.6 | 12.6 (11.9-13.3) | 348 | 31.9 | 28.6 (25.3-31.8) |
| *Ill-defined/Sec.* | 1,716 | 8.1 | 15.7 (15-16.5) | 402 | 36.8 | 24.6 (22-27.2) |
| *Ovary* | 2,334 | 11.1 | 20.8 (19.9-21.7) | 306 | 28.0 | 24.3 (21.3-27.2) |
| *Thyroid/Endocrine* | 1,746 | 8.3 | 11.7 (11.1-12.3) | 234 | 21.4 | 23 (19.9-26.1) |
| *Leukaemia* | 2,079 | 9.9 | 16.7 (15.9-17.5) | 306 | 28.0 | 22.4 (19.7-25.1) |
| *Stomach* | 1,032 | 4.9 | 9 (8.5-9.6) | 273 | 25.0 | 19.9 (17.4-22.5) |
| *Liver* | 636 | 3.0 | 5.7 (5.3-6.2) | 237 | 21.7 | 16.1 (13.9-18.4) |
| *Head/Neck* | 1,329 | 6.3 | 12 (11.4-12.7) | 204 | 18.7 | 16 (13.6-18.4) |
| *Myeloma* | 1,131 | 5.4 | 10.9 (10.2-11.5) | 201 | 18.4 | 14.5 (12.3-16.6) |
| *Eye/Brain/CNS* | 1,341 | 6.4 | 10.7 (10.1-11.3) | 174 | 15.9 | 13.2 (11.1-15.4) |
| *Cervix* | 1,332 | 6.3 | 8.3 (7.8-8.9) | 114 | 10.4 | 11.2 (9.1-13.4) |
| *Gall/Biliary* | 552 | 2.6 | 5.2 (4.8-5.7) | 156 | 14.3 | 10.7 (8.8-12.5) |
| *Bladder* | 825 | 3.9 | 7.7 (7.1-8.2) | 168 | 15.4 | 10.2 (8.5-11.8) |
| *Meso/Soft Tissue* | 747 | 3.5 | 6.1 (5.7-6.6) | 108 | 9.9 | 8.7 (6.9-10.5) |
| *Oesophageal* | 738 | 3.5 | 7 (6.5-7.5) | 120 | 11.0 | 7.2 (5.8-8.6) |

b)

|  | **Male** | | | | | |
| --- | --- | --- | --- | --- | --- | --- |
|  | **Without Diabetes** | | | **With Diabetes** | | |
|  |  | Cancer Rate (n/100,000 PY) | |  | Cancer Rate (n/100,000 PY) | |
|  | *n* | *Crude* | *Age Std.* | *n* | *Crude* | *Age Std.* |
| **Total** | 91,260 | 442 | 1014.6 (1007.8-1021.3) | 17,343 | 1,557 | 1156.3 (1138-1174.6) |
|  |  |  |  |  |  |  |
| **By Cancer** |  |  |  |  |  |  |
| *Prostate* | 26,694 | 129.2 | 321 (317.2-324.9) | 4,068 | 365.1 | 273.3 (264.5-282.1) |
| *Colorectal* | 12,180 | 59.0 | 138 (135.5-140.5) | 2,607 | 234.0 | 167 (160.2-173.8) |
| *Lung* | 8,232 | 39.8 | 95.9 (93.8-98) | 2,067 | 185.5 | 132.1 (126.1-138.1) |
| *Melanoma* | 10,728 | 51.9 | 114.7 (112.5-117) | 1,425 | 127.9 | 92.6 (87.4-97.7) |
| *Kidney* | 2,721 | 13.2 | 29.7 (28.5-30.8) | 660 | 59.2 | 48.6 (44.6-52.5) |
| *Liver* | 1,311 | 6.3 | 14.3 (13.5-15.1) | 678 | 60.9 | 48.4 (44.5-52.2) |
| *Pancreas* | 1,734 | 8.4 | 19.8 (18.9-20.7) | 693 | 62.2 | 45.8 (42.2-49.4) |
| *NH Lymphoma* | 3,615 | 17.5 | 38 (36.7-39.3) | 570 | 51.2 | 39.3 (35.9-42.8) |
| *Stomach* | 1,773 | 8.6 | 19.6 (18.7-20.5) | 513 | 46.0 | 34.9 (31.7-38.1) |
| *Leukaemia* | 3,105 | 15.0 | 30.2 (29.1-31.4) | 513 | 46.0 | 34.7 (31.5-37.9) |
| *Head/Neck* | 3,033 | 14.7 | 32.4 (31.2-33.6) | 456 | 40.9 | 33 (29.8-36.2) |
| *Bladder* | 2,163 | 10.5 | 25.1 (24-26.2) | 477 | 42.8 | 27.6 (25-30.2) |
| *Ill-defined/Sec.* | 1,593 | 7.7 | 18.1 (17.2-19) | 447 | 40.1 | 27.2 (24.5-29.8) |
| *Oesophageal* | 1,512 | 7.3 | 17.6 (16.7-18.5) | 339 | 30.4 | 22.1 (19.6-24.6) |
| *Myeloma* | 1,596 | 7.7 | 18.1 (17.2-19) | 330 | 29.6 | 21.9 (19.4-24.4) |
| *Eye/Brain/CNS* | 1,773 | 8.6 | 16.4 (15.6-17.3) | 234 | 21.0 | 18.3 (15.8-20.8) |
| *Meso/Soft Tissue* | 1,350 | 6.5 | 13.9 (13.1-14.7) | 219 | 19.7 | 15.3 (13.1-17.5) |
| *Thyroid/Endocrine* | 681 | 3.3 | 5.6 (5.2-6.1) | 135 | 12.1 | 12.2 (10-14.4) |
| *Gall/Biliary* | 414 | 2.0 | 4.8 (4.3-5.3) | 144 | 12.9 | 9.6 (7.9-11.3) |
| *Testis* | 1,416 | 6.9 | 5.9 (5.5-6.3) | 45 | 4.0 | 5.3 (3.6-6.9) |
